# The Ecology of Beech Bark Beetle Mycobiomes: Similar Fungal Composition Despite Host, Elevation and Site Gradients

**DOI:** 10.1007/s00248-026-02910-3

**Published:** 2026-11-04

**Authors:** Lena Carlson, Vienna Kowallik, Katrin Hübscher, Lena Rabenhofer, Kaya Schernickau, Peter H. W. Biedermann

**Affiliations:** 1 Chair of Forest Entomology and Forest Protection, University of Freiburg, Freiburg, Germany; 2 Department of Animal Ecology and Tropical Biology, University of Würzburg, BiocenterWürzburg, Germany; 3 Future Forests Cluster of Excellence, University of Freiburg, Freiburg, Germany

**Keywords:** Mycobiome, Fungi, Bark beetle, Forest ecology, Forest heterogeneity, *Ernoporicus fagi*, *Taphrorychus bicolor*, BETA-FOR

## Abstract

Bark beetles form varying associations with fungi, yet the mycobiomes of many deciduous phloem-feeding species are poorly studied and their ecological significance remains underexplored. Here, we used ITS2 metabarcoding to characterize the fungal communities of the beech bark beetles *Ernoporicus fagi* and *Taphrorychus bicolor* collected from 61 forest patches across six regions in Germany. Despite sampling two beetle species across an elevational gradient, experimental forest management treatments, and six regions, beetle species identity, treatment, and elevation explained little variation in beetle mycobiomes, and region showed only a weak effect that disappeared under closer inspection. However, we found mycobiome composition varied more consistently at the finer scale of individual forest sites, and geographic distance between patches had a weak but significant effect, suggesting that the fungal communities these bark beetles carry are shaped by local, patch-level factors rather than broader regional or host-related factors. The mycobiomes of both beetle species were largely similar and dominated by ubiquitous saprotrophs and yeasts.

**Supplementary Information:**

The online version contains supplementary material available at https://doi.org/10.1007/s00248-026-02910-3.

## Introduction

With climate change driving more frequent bark beetle outbreaks and increasingly severe impacts on forests, interest in bark beetle-fungus associations has grown [25, 44, 45]. While some conifer-associated bark beetle-fungus systems (e.g., *Dendroctonus*, *Ips* species) are well studied, the fungal communities of many deciduous phloem-feeding species and their variability across environmental gradients are still poorly understood. Two such examples are the mycobiomes of the beech bark beetles *Ernoporicus fagi* (F., 1778) and *Taphrorychus bicolor* (Hbst., 1793). While most bark beetle species are associated with fungi, the degree and type of association vary greatly [12]. Many ambrosia beetles as well as a few bark beetles (e.g., some *Dendroctonus* and *Ips* spp.), carry the spores of the fungi they feed on in specialized organs called ‘mycetangia’ and form obligate mutualistic relationships with specific fungal species [19, 22, 35]. However, most phloem-feeding bark beetles typically have been associated with an array of fungal species in more facultative relationships [33]. Some bark and ambrosia beetle mycobiomes are relatively well studied, such as the aggressive primary bark beetle *Ips typographus* (L., 1758) (e.g., [30]) and the ambrosia beetle *Xyleborinus saxesenii* (Ratzeburg, 1837) (e.g., [6]). Symbiont communities can include mutualists, commensals, and antagonists [13, 15]. Facultative fungal associations, where beetles associate with locally available organisms, appear to be a successful strategy for many Scolytinae-fungus associations [35]. How those associations are shaped by trans-generational (vertical) transmission, tree-host factors, climatic factors (e.g., temperature), forest management and landscape-scale gradients remains poorly understood, even for the primary model beetles such as *Ips typographus* [30] and particularly so for less-studied deciduous phloem-feeding species. More heterogeneous forest environments, for example, may be related to higher fungal symbiont diversities [43], possibly due to greater structural variations (e.g., varying deadwood amounts, canopy openness leading to more microclimate variation) leading to increases in the range of fungal niches available for beetles to encounter.

In this multi-region field study, we focused on the much less studied mycobiomes of the phloem-feeding bark beetles *E. fagi* and *T. bicolor* (Coleoptera: Curculionidae: Scolytinae). *Ernoporicus fagi* and *T. bicolor* breed and feed in the phloem of European beech (*Fagus sylvatica*) and rarely in hornbeam (*Carpinus betulus*) and oaks (*Quercus* spp.). While *E. fagi* prefers the branches and twigs of decaying trees, *T. bicolor* typically attacks the trunks of stressed and dying trees [27]. Adults are between 1.5–1.8 mm and 1.6–2.3 mm in length, respectively [8]. Although both species typically have two generations a year and co-occur across Central European beech forests, they also differ in mating system, gallery structure and phenology: *E. fagi* flies in May and July, and *T. bicolor* flies earlier, from March to June [8]. *E. fagi* is monogamous with irregular and longitudinal galleries bored by single females, while *T. bicolor* is polygynous with multiple-females boring star-shaped galleries [8, 27]. Known fungal associates of *T. bicolor* are *Graphium* sp., *Graphium cf. penicilloides, Ophiostoma* sp.*, Ophiostoma quercus, Ophiostoma pseudokarelicum, Geosmithia* spp. and *Leptographium* sp. [13, 14, 40] while *E. fagi*’s documented fungal associations are limited to some *Geosmithia* species [17, 40]. However, these previous studies relied entirely on culturing methods rather than DNA metabarcoding to characterize the fungal communities. Hence, the mycobiomes of both *E. fagi* and *T. bicolor* overall remain poorly characterized. Yet understanding them is relevant for understanding their biology as well as their interactions with the abiotic and biotic environment which includes the tree host. This can help predict how these insect-fungus symbioses may respond to ongoing environmental change and affect their host trees in the future by showing which fungi these beetles carry, serving as a baseline for possible future fungal community shifts. European beech is becoming increasingly stressed and susceptible to drought, in turn making it more susceptible to attack by bark beetles and fungal pathogens [18].

To gain a better understanding of the identity and variability of fungal associates of *E. fagi* and *T. bicolor* and potential effects by the local environments, we distributed beech logs on experimental forest plots in different regions and varying environmental conditions [24]. After three months of colonization followed by breeding within these logs, we collected emerging bark beetles with emergence traps and used internal transcribed spacer 2 (ITS2) metabarcoding to characterize the beetles’ mycobiomes to genus and species level. Throughout this study, we define mycobiome as the fungal community detected by ITS2 metabarcoding from whole crushed beetles. Since beetles were analyzed without surface sterilization, their mycobiomes are expected to reflect both internal and external fungi. With the mycobiome data we tested the following hypotheses:

- *Hypothesis 1:* Mycobiome composition differs between the beech bark beetles *E. fagi* and *T. bicolor* because they colonize different host material with different microhabitat conditions.
- *Hypothesis 2:* Mycobiome composition shifts along an elevational gradient due to temperature-driven filtering of beetle and fungus associations prior to dispersal [34].
- *Hypothesis 3:*Beetle mycobiomes in heterogeneous forest patches show greater diversity and are more distinct than in more homogeneous production forest patches, due to higher niche availability for fungi in the surrounding environment.
- *Hypothesis 4: *Overall mycobiome composition differs by region, as broad-scale climatic and structural differences among regions influence beetle fungal mycobiome compositions.

We then characterized dominant and ecologically-relevant fungal species by prevalence and relative abundance and discuss their potential ecological functions.

## Materials and Methods

### Experimental Design

**Fig. 1:**
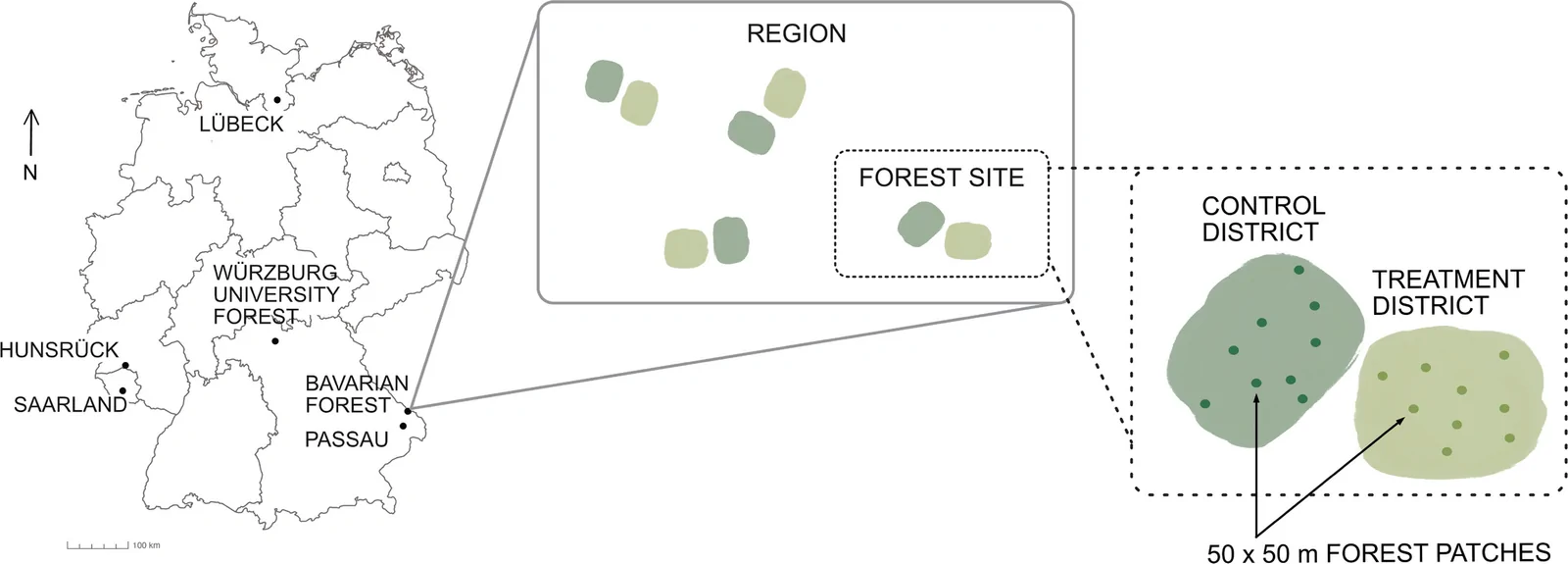
Beetles were collected from logs placed on 61 forest patches (structurally heterogeneous treatment patches and more homogeneous control patches) in production forests across six regions in Germany

This study was conducted on a subset of 61 forest patches (50 m × 50 m) within the BETA-FOR experimental framework across six regions (Würzburg University Forest, Bavarian Forest, Passau Forest, Hunsrück Forest, Saarland Forest and Lübeck Forest) in Germany [24]. Within each region, patches are nested within forest sites, each containing control and treatment patches. Deadwood and canopy manipulation treatments were implemented between winter 2015 and winter 2018, creating a more heterogeneous forest structure on treatment forest patches as compared to control forest patches (Fig. 1). For a detailed description of the full BETA-FOR design, see Müller et al. [24].

Here, we characterize the fungal community composition of a subset of two beech bark beetles collected from the 61 forest patches on which placed beech logs were naturally colonized by the target beetle species. All forest stands were production forests dominated by European beech (*Fagus sylvatica*), or made up of diverse mixtures dominated by native broadleaf species (primarily *Quercus*, *Fraxinus*, *Acer*, *Carpinus*, and *Tilia* spp.), with some conifer species such as Norway spruce (*Picea abies*) and Scots pine (*Pinus sylvestris*).

To sample beetles, freshly cut beech logs (approximately 10 cm in diameter and 50 cm long) were sourced from trees in the surrounding forest area of each region and were suspended on the forest patches at 1 m height, one beech log per patch, from mid-April to early July to allow colonization by bark beetles. Logs were hung at Bavarian Forest, Passau, and University Forest sites in 2022, and in 2023 at Hunsrück, Saarland, and Lübeck sites. Across the six regions, 157 beetles (66 *E. fagi*, 91 T*. bicolor*) were collected from 61 forest patches, each with a single suspended beech log for colonization. Since individual beetles did not consistently yield sufficient biomass for DNA extraction, specimens from the same log were pooled by species wherever more than one was available, resulting in 83 samples (41 *E. fagi*, 42 T*. bicolor*; 1–3 beetles per sample: 41 samples with 1 beetle, 10 with 2, 32 with 3). On four patches, both beetle species emerged from the same log, resulting in paired same-log samples for the two species. We tested whether fungal community composition differed between samples containing multiple pooled beetles and samples containing a single beetle. These did not differ significantly, so we analyzed all samples together. After exposure, logs were transported to the University of Freiburg, sealed with paraffin to prevent drying, and placed in emergence traps (aerated PVC pipes with eclectors). Traps were emptied weekly over a period of one year. Emerged beetles were identified morphologically to species level using Grüne [8] and the online resource Coleonet (https://www.coleonet.de/coleo/texte/curculionidae.htm), with Sanger sequencing of the COI barcoding region for beetle identification verification where necessary. Primers dgHCO2198 (5′-TAAACTTCAGGGTGACCAAARAAYCA-3′) and mlCOIintF (5′-GGWACWGGWTGAACWGTWTAYCCYCC-3′) were amplified with PCR conditions of 3 min at 95 °C, followed by 35 cycles of 30 s at 95 °C, 30 s at 55 °C and 50 s at 72 °C, and 5 min at 72 °C (10 °C hold). Live beetles were collected, stored at − 20 °C and transferred to a shield buffer shortly before DNA extraction.

To examine the full external and internal fungal community of the emerged beetles, DNA was extracted from full beetles using the ZymoBIOMICS™ 96 MagBead DNA Kit and according to the accompanying protocol. Together with the beetle samples, negative, blank, and no-template controls (six in total) as well as three mock community samples (ZymoBIOMICS Microbial Community Standard) were extracted and processed side-by-side with the beetle samples to control for laboratory and cross contamination and sequencing performance. We sent the DNA from the 83 beetle samples, plus the nine controls to the Institute of Clinical Molecular Biology (IKMB) where library preparation, sequencing, demultiplexing and primer removal was performed [32, 43]. For fungal communities the internal transcribed spacer 2 (ITS2) was sequenced with a 250 bp paired-end strategy on one sequencing chip on a MiSeq. ITS2 amplicons were generated using fungal primers 5.8 S-Fun_1 (5′-AACTTTYRRCAAYGGATCWCT-3′) and ITS4-mod (5′-AGCCTCCGCTTATTGATATGCTTAART-3′). Samples were multiplexed following the protocol of Taylor et al. [41], and demultiplexed after sequencing. PCR products were normalized using the SequalPrep Normalization Plate Kit (Thermo Fisher Scientific, Waltham, MA, USA) and pooled prior to sequencing. Since custom primers were used, primer sequences were removed during demultiplexing rather than in a separate trimming step, following the IKMB amplicon processing workflow ([32], https://github.com/mruehlemann/ikmb_amplicon_processing).

All downstream processing was carried out in R v.4.2.2 using DADA2 v1.26.0 [2] following the IKMB amplicon processing workflow [32] with slight modifications. To find the best processing strategy for our data set, we compared a forward read only approach against a merge only approach with non-overlapping pairs being discarded as well as a mix approach where the non-merged reads were concatenated in an additional step concatenated. We realized that the merging-only strategy resulted in the loss of 62 fungal genera compared to the forward-only approach. The mixed strategy was much more similar to the forward strategy, however, as concatenation is known to introduce ASV artifacts, we finally decided to use the forward only processing to avoid length-related loss of taxa. The first 5 bp from each read, low-quality bases were trimmed and forward reads were shortened to 248 bp. Reads containing any ambiguous base (maxN = 0) and those exceeding the allowed expected error rates (maxEE = 3) or having a quality score below the truncation threshold (truncQ = 3) were discarded. Error models were estimated using the learnErrors function with the default number of bases in a randomized order. Identical reads were collapsed to unique sequences with derepFastq. With these learned error models, the DADA2 core algorithm was applied to the forward reads.

A table of amplicon sequence variants (ASVs) per sample was created and potential chimeric ASVs were identified and removed using the consensus method. ASVs were classified against the UNITE fungal ITS reference database [1] dynamic release 2025‑02‑19 dev. version, with *assignTaxonomy* while considering the reverse complement of the query.

Fungal ASVs were subsequently analyzed in R version 4.4.3 (2025–02–28) with the *phyloseq* [23], *iNext* [11], *vegan* [28] and *ComplexHeatmap* [9] packages. After removing non-fungal reads as well as singletons and samples with a read number below 1000, 63 samples were retained for further analysis. A threshold of 1000 was chosen based on iNEXT rarefaction curves, which showed that the dominant components of the mycobiome (*q* = 1, *q* = 2) were well sampled at this depth (Figure S1).

We used measured elevation values (m a.s.l) for each forest patch. Gaps were filled with digital elevation model (DEM 200) values in the few instances where on-patch measurements were unavailable. X and Y coordinates (WGS84) were used for the locations of forest patches.

### Statistical Analysis

Four negative controls, two blanks and three mock positive controls were processed through the same pipeline as the rest of the samples. Six contaminant ASVs were identified using the *decontam* package with a threshold of 0.5 [4] and removed before further analysis. To test for potential trap effects (i.e., whether individual PVC pipe traps systematically shaped beetle mycobiome composition), we compared Bray–Curtis dissimilarity between within-trap sample pairs and between-trap sample pairs across the full set of emerged beetle species (not just the subset of emerged beetles studied here) using a Wilcoxon test.

The data was rarefied and extrapolated using iNext curves and Hill number orders of q, where q = 0 (treating all ASVs equally) is ASV richness, q = 1 (weights ASVs relative to their abundance) is Hill-Shannon, q = 2 (weights most abundant ASV, downscaling the rarer ones) is Hill-Simpson. Then, alpha diversity was quantified using Shannon diversity expressed as a Hill number (q = 1). This transforms the Shannon index into an effective number of species. To confirm that results were not affected by rarefaction, all PERMANOVA analyses were repeated on proportion normalized data. We used the Hill-Shannon diversity index (q = 1) in all analyses. Hill-Shannon diversity is expressed as the effective number of equally common species, so values are directly comparable across groups. Raw Shannon and Simpson values are provided in Figure S2 in the supplements for comparison with studies not reporting Hill numbers. To characterize the distribution of key fungal taxa, we calculated prevalence (the percentage of beetle samples in which a specific fungal taxon was detected) and mean relative abundance (the per-sample proportion of sequence reads assigned to a taxon averaged across all samples). When interpreting results, it should also be kept in mind that fungal ITS reads vary substantially between taxa and relative abundance from ITS metabarcoding is semi-quantitative [42]. For visualization (Fig. 5), the three most dominant genera (*Cladosporium, Penicillium, Aspergillus*) are shown at genus level due to their high within-genus diversity and *Ophiostoma* and *Beauveria* are also shown at genus level, since the single detected ASV could not be resolved to species. All other taxa are shown at species level. Finally, we tested for indicator species associated with beetle hosts using multilevel pattern analysis [5], with 9999 permutations and Benjamini–Hochberg correction for multiple testing.

Since some regions in this design contain only one to two sampled forest sites, region and forest site are partially confounded. We added forest site as a spatial grouping variable to test H4 without this pseudo replication in addition to testing the significance of region. To test for spatial autocorrelation independent of the region and forest site groupings above, we used multiple regression on distance matrices (MRM) with geographic distance between patches as a predictor of mycobiome dissimilarity. To characterize the bark beetle mycobiome we use two analyses: its composition (beta diversity), tested using PERMANOVA and MRM, and its diversity (alpha diversity), tested using Hill numbers.

## Results

Pooling beetles did not affect fungal community composition (R^2^ = 0.012, *p* = 0.792). Sequenced negative controls and blanks had a lower read depth on average (raw reads: min = 1; mean = 1554; max = 7232) than samples (after filtering and before rarefaction: min = 1003; mean = 6170; max = 11,305), and mock positive controls contained the expected fungal genera. Six ASVs were labeled as contaminants and removed (0.11% of reads) and did not affect the main PERMANOVA results. There was no significant trap effect (medians 0.911 within-trap vs. 0.874 between-trap, Wilcoxon *p* = 0.933).

iNEXT curves showed that the dominant components of the mycobiome (*q* = 1, Shannon; *q* = 2, inverse Simpson) were well sampled, although total ASV richness was under sampled (*q* = 0) (see Figure S1 in supplements). PERMANOVA results were almost identical when analyses were repeated on proportion-normalized data (Table S1 in supplements).

**Fig. 2:**
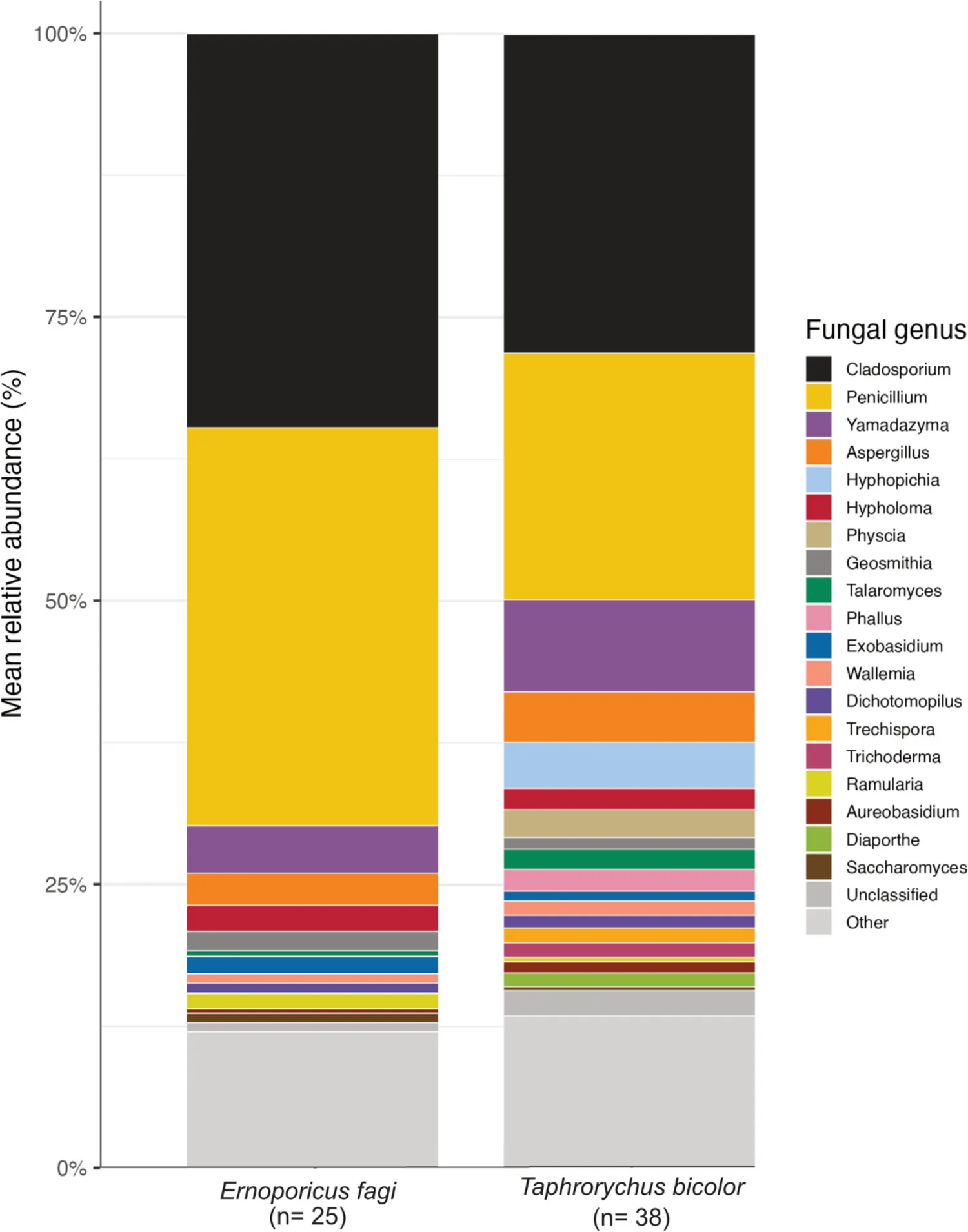
Fungal composition comparison between *E. fagi* (*n* = 25) and *T. bicolor* (*n* = 38) based on ITS amplicon sequencing, showing mean relative abundance of the 20 most abundant fungal genera ranked by mean relative abundance across all samples. “Other” includes all genera outside the top 20 by mean relative abundance

Pooling fungal composition by beetle species shows that, at the genus level, ratios differ slightly but overall compositions are similar between *E. fagi* and *T. bicolor*. The dominant genera for both beetle species are *Cladosporium*, *Penicillium*, *Yamadazyma* and *Aspergillus.* Among the more abundant genera, *Hyphopichia*, *Physcia*, *Phallus,* and *Trechispora* occur only in *T. bicolor* samples. Mean relative abundance of *Cladosporium* and *Penicillium* was noticeably higher in *E. fagi* samples, while *Yamadazyma* and *Aspergillus* were higher in *T. bicolor* (Fig. 2).

**Fig. 3:**
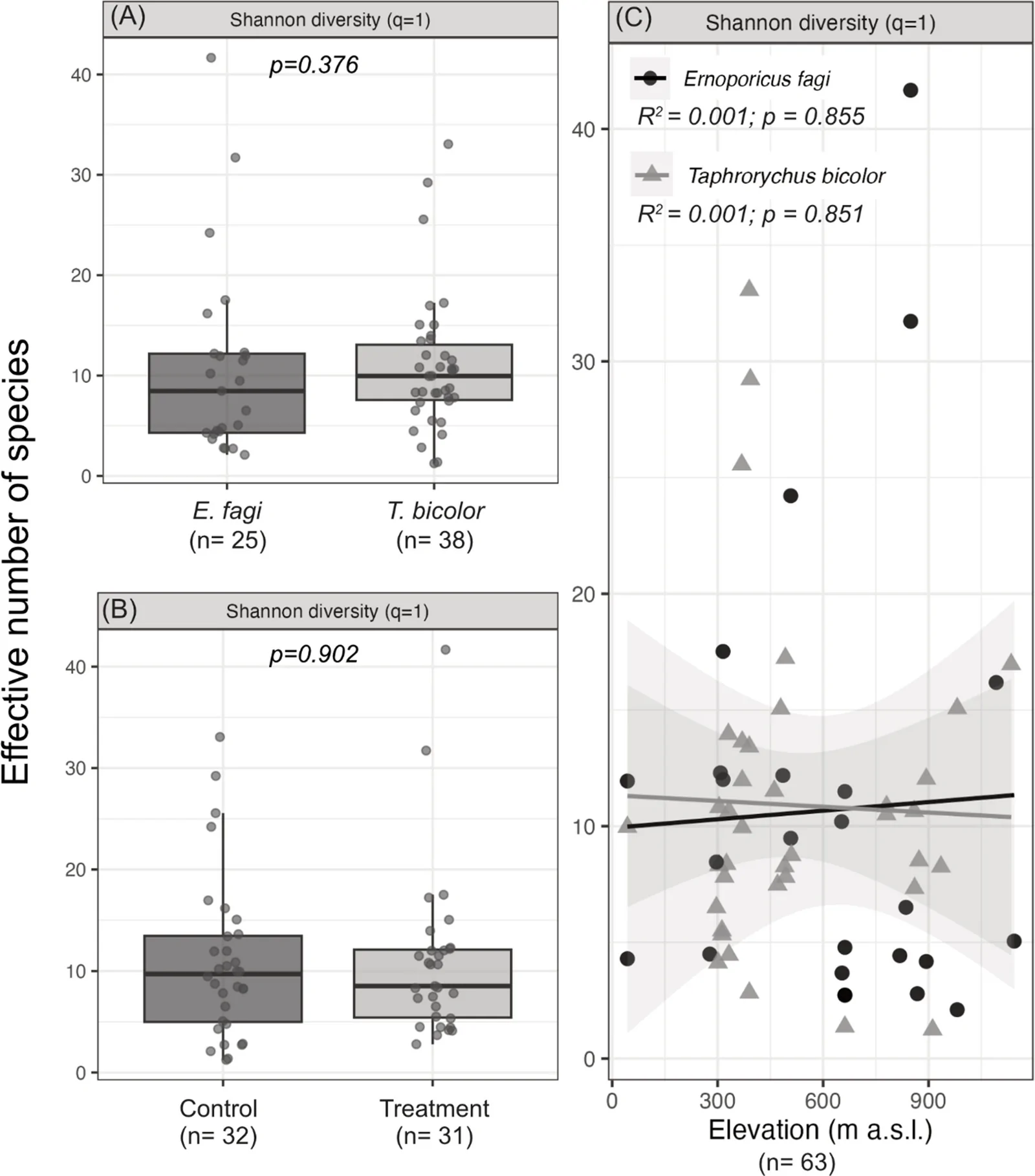
Shannon diversity (Hill number *q* = 1; effective number of species) of fungal communities associated with *E. fagi* and *T. bicolor*. Boxplots show no significant differences in alpha diversity (A) between beetle species (*p* = 0.376) or (B) between control and treatment forest patches (*p* = 0.902). Linear models (C) show no relationship between Shannon diversity and elevation for either species (R^2^ ≤ 0.001, *p* ≥ 0.851)

Contrary to H1, mycobiome composition did not differ significantly between beetle species (PERMANOVA: *p* = 0.1185 in the full model, *p* = 0.0521 species-only) (Table S5), nor did alpha diversity (Kruskal–Wallis, *p* = 0.085 for ASV richness; Fig. 3A). We found no support that elevation significantly affects alpha diversity of either beetle’s mycobiome (Shannon diversity: *E. fagi* *p* = 0.855, *T. bicolor* *p* = 0.851; ASV richness: *E. fagi* *p* = 0.378, *T. bicolor* *p* = 0.807), rejecting H2 (Fig. 3C). We found no evidence that treatments shape the mycobiomes of *E. fagi* and *T. bicolor*. Neither alpha diversity (*p* = 0.902) nor community composition (PERMANOVA, R^2^ = 0.0110, *p* = 0.8435) differed between treatment and control patches, rejecting H3 (Fig. 3B). Although the sampling design is unbalanced across regions and forest sites, the non-significant effects of beetle species, elevation and treatment are robust in this dataset because the associated effect sizes are close to zero and ordinations show no trends along these gradients. Any true effects of these variables on mycobiome composition are likely smaller than the unexplained variation in the dataset.

**Fig. 4:**
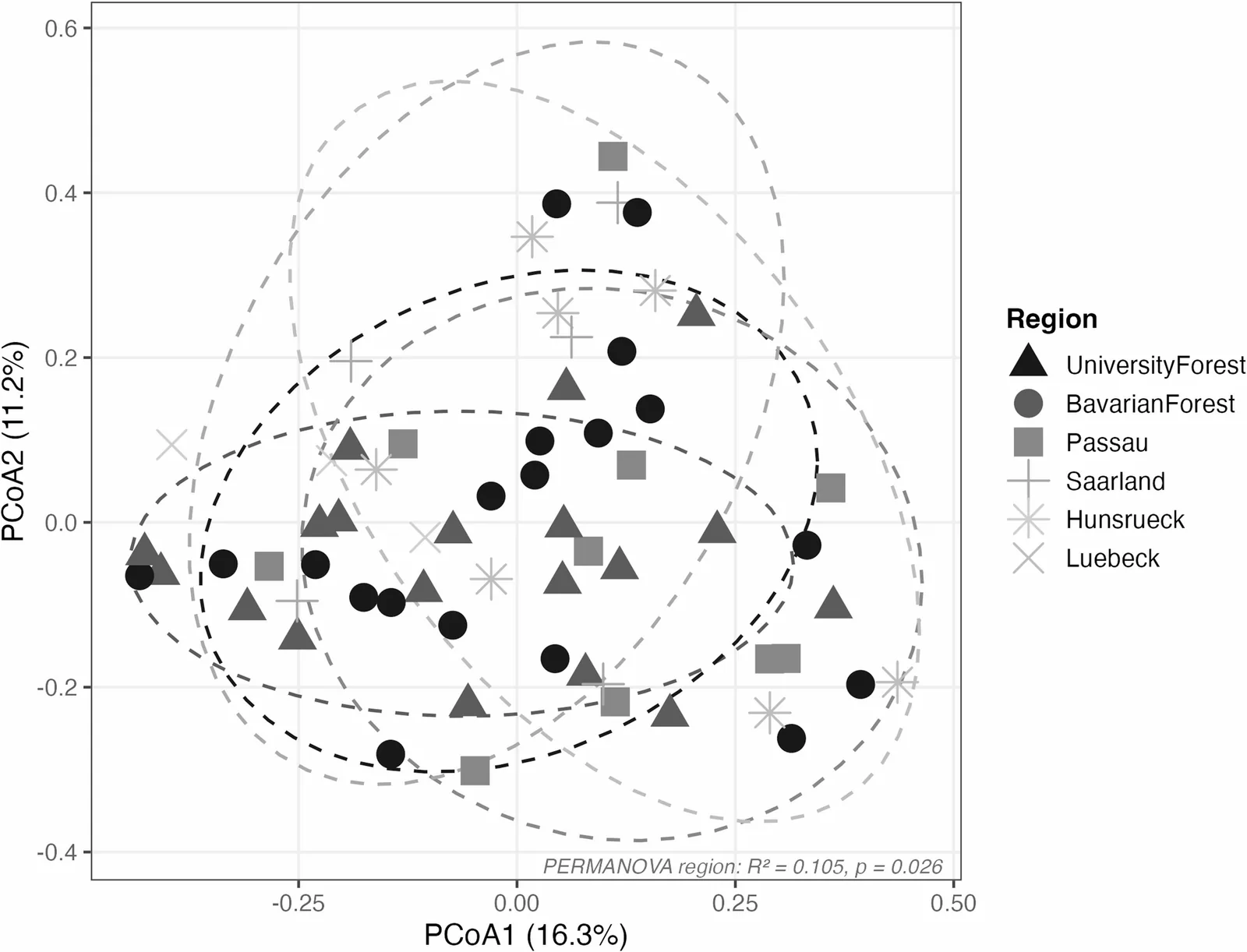
Principal Coordinate Analysis (PCoA) of fungal community composition for samples by region. Regions with too few samples do not have ellipses (e.g., Luebeck)

Region initially showed a weak but significant effect on mycobiome composition (Fig. 4), with PERMANOVA results of R^2^ = 0.105 and *p* = 0.026. This regional effect disappeared after two sensitivity checks (Table S3). First, when Hunsrück and Lübeck (regions with only 1–2 samples) were excluded, region was no longer significant among the remaining four regions (R^2^ = 0.071, *p* = 0.094). Second, a direct pairwise comparison between the two best-sampled regions, University Forest and Bavarian Forest, also showed no difference (R^2^ = 0.027, *p* = 0.435). This finding indicates that region, as tested at this broad scale, is not a robust driver of mycobiome composition, and leads us to reject H4. However, because many regions in this design contain only one or two sampled forest sites, a region-level test risks pseudoreplication and may obscure spatial structure at a finer scale. We therefore additionally tested forest site as the spatial unit. Mycobiome composition was significantly structured by forest site across the full dataset (PERMANOVA, R^2^ = 0.208, *p* = 0.005), and this result held when tested independently of region-site confounding within the two most completely sampled regions (R^2^ = 0.174, *p* = 0.028). This suggests that spatial structuring of mycobiome composition exists, but at a finer resolution than the regional grouping tested in H4. Our MRM test for spatial autocorrelation showed geographic distance was a significant predictor (coefficient = 0.0100, *p* = 0.011) explaining 2.2% of variance overall (R^2^ = 0.022, *p* = 0.033) (Table S4). The homogeneity of the multivariate dispersion did not differ significantly for any grouping variable tested (all *p* > 0.18), indicating that the PERMANOVA results reflect true differences between groups rather than differences in within-group variability.

**Fig. 5:**
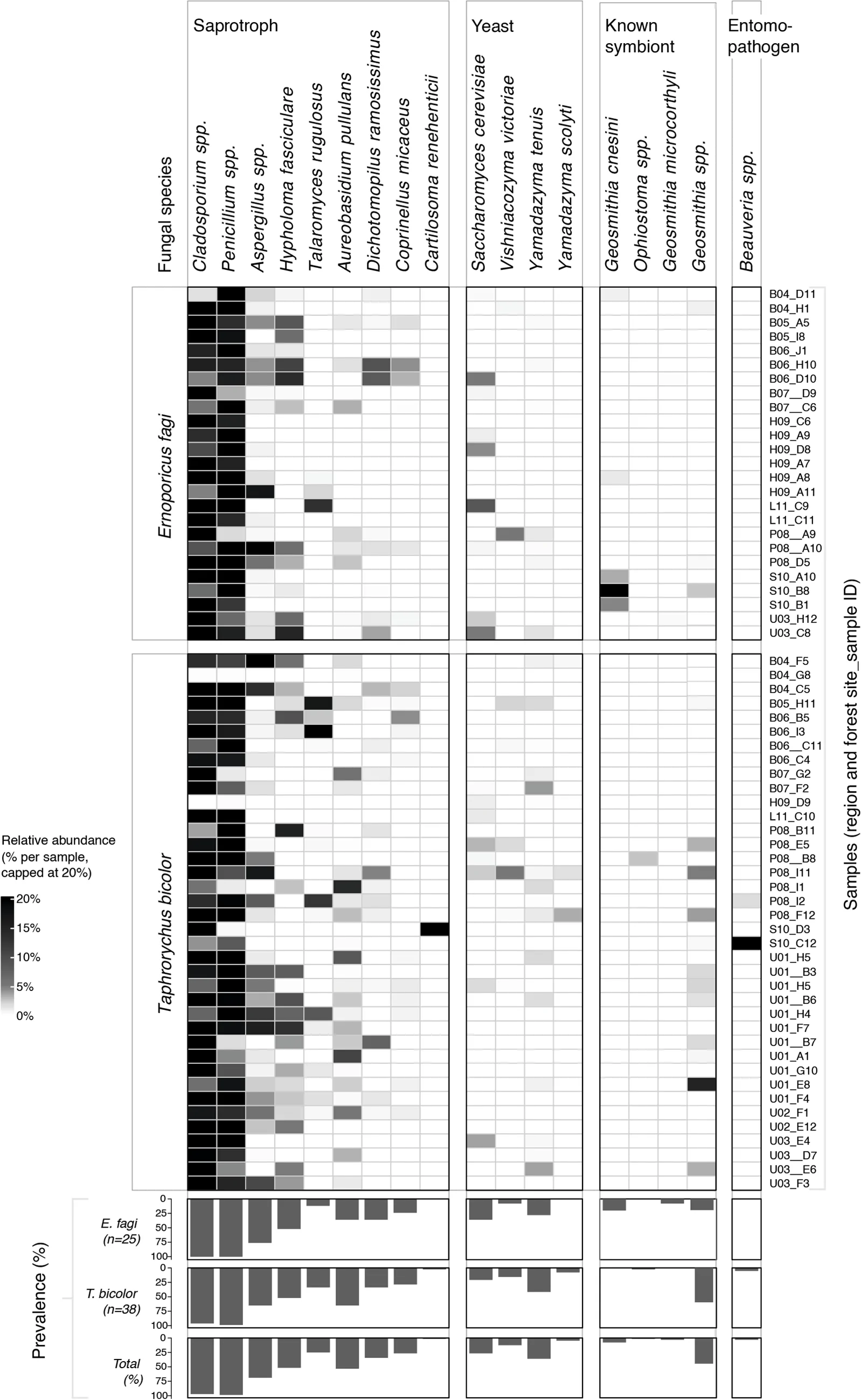
Heatmap of key fungal species in *Ernoporicus fagi* and *Taphrorychus bicolor* mycobiomes grouped by ecological categories (saprotrophs, yeasts, known symbionts and entomopathogens), with variation in relative abundance and prevalence across beetle samples. Samples are ordered by region and forest site followed by sample ID. *Cladosporium*, *Penicillium*, and *Aspergillus* are shown to genus level because of high within-genus diversity. *Ophiostoma*, *Beauveria*, and *Geosmithia* spp. are shown to genus level because the ASVs could not be resolved to species

We then examined the occurrence and abundance of fungal genera associated with *E. fagi* and *T. bicolor.* The indicator species analysis shows no fungal ASVs were significantly associated with either beetle species after FDR correction (all p. adj ≥ 0.05), consistent with the absence of strong compositional differentiation between hosts. Nevertheless, confirming what we already indicated in Fig. 5, the saprotrophs *Cladosporium*, *Penicillium* and *Aspergillus* genera are most prevalent and abundant across both beetle species, with *Cladosporium* and *Penicillium* genera present in close to all samples. *Aspergillus* occurs in approximately 75% of all samples. *Hypholoma fasciculare* has a high prevalence across samples and in both beetle species. *Talaromyces rugulosus* is found mostly with *T. bicolor*, where it has a prevalence of over 25%. Prevalent across both species, *Aureobasidium pullulans* is slightly more prevalent and abundant with *T. bicolor* than *E. fagi*. *Dichotomopilus ramosissimus* is less abundant but prevalent across both species. *Coprinellus micaceus* has a greater prevalence with *T. bicolor*, but is found with both species. *Cartilosoma renehenticii* was detected with high relative abundance in a single *T. bicolor* sample but was otherwise rare. Of the yeasts, both *Saccharomyces cerevisiae* and *Vishniacozyma victoriae* are represented in over 25% of samples for both species. *Yamadazyma tenuis* is less prevalent (less than 25%) with both species. *Yamadazyma scolyti* is only found in two samples in *T. bicolor* and absent from samples of *E. fagi.* Of known beetle symbionts in the genera *Geosmithia* and *Ophiostoma*, *Geosmithia* was detected in 23 samples (826 reads total) across both beetle species and most regions, though the species *G. cnesini* occurred predominantly in three *E. fagi* samples in the Saarland region, while *G. microcorthyli* was detected in trace amounts. *Geosmithia* spp. reads unresolved to species were more broadly distributed, occurring predominantly across several *T. bicolor* University Forest and Passau samples (Fig. 5). *Ophiostoma* spp. was detected only once, in a single *T. bicolor* sample. *Beauveria* spp., a known bark beetle entomopathogen, was detected in two *T. bicolor* samples.

## Discussion

This study set out to gain a better understanding of the underexplored fungal associates of the bark beetles *E. fagi* and *T. bicolor*. First, we tested whether beetle host species significantly influence mycobiome composition. Then we tested whether mycobiome composition shifts along an elevational gradient and whether structurally heterogeneous forest patches lead to greater diversity in fungal mycobiomes than more homogenous forest patches and whether the region affects mycobiome composition. Finally, we characterized dominant and ecologically relevant fungal taxa by prevalence and relative abundance and discuss their potential ecological roles. Beetle host species, elevation, and treatment did not significantly shape mycobiome composition, and the regional effect we detected was not robust. However, mycobiome composition was significantly structured at the finer scale of individual forest sites, and geographic distance between patches also had a significant effect, indicating that spatial structure exists at a finer resolution than region alone captures. Both *E. fagi* and *T. bicolor* share broadly similar mycobiomes dominated by ubiquitous saprotrophs, with only a few host-specific exceptions.

We used a combination of occurrence across samples within a species and relative abundance within each individual sample to assess fungal associations. Conifer bark beetle systems show that specific fungal symbionts can be important for beetle development and fitness. For example, they can be involved in detoxifying host tree defense chemicals [10] and making nutrients such as amino acids and vitamin B6 available as with *Ips typographus* [3] or making nitrogen and phosphorus available to primary and secondary *Dendroctonus* and *Ips* [36, 37]. For angiosperm colonizing bark beetles, far less is known. Documented fungal associations of *E. fagi* and *T. bicolor* come from culture-based work and are limited to a handful of genera [13, 14, 17, 40]. Our data extend this picture in two ways. They show that the mycobiomes of both species are dominated by ubiquitous saprotrophs and yeasts rather than by the ophiostomatoid fungi that characterize many conifer systems, and they show that these assemblages remain broadly similar across large-scale climatic gradients. Any effects of fungal symbionts on angiosperm colonizing beetles remain uncertain. However, it is likely that many of the same factors that influence the distributions and prevalence of fungi with angiosperm colonizing beetles may still be at play including the chemical composition of the tree environment and abiotic factors including moisture, temperature and season, which may filter which mutualists beetles carry [33, 34, 38].

Contrary to H1, we found no clear host-species separation in fungal mycobiomes. In fact, beetle species identity explained only a small amount of compositional variation. This is supported by our indicator species analysis, which found no fungal ASVs significantly associated with either beetle host after correcting for multiple testing, further supporting the absence of strong host-specific associations. The overlapping mycobiomes of *E. fagi* and *T. bicolor* and the weak effects of forest management and elevation are consistent with the idea that most phloem-feeding bark beetles colonizing angiosperms do not have obligate, species-specific core symbionts, but rather acquire fungi from the substrate and exchange many taxa horizontally and between beetle species [35]. In these systems, broadly distributed environmental or host-tree specific fungal pools and opportunistic acquisition are expected to outweigh strong host filtering [35]. This suggests that subtle, fine-scale processes at the log and host-tree level (e.g., individual variation in host-tree defenses, water content etc.) influence relative abundances rather than creating strong regional differentiation.

Špoula et al. [39] showed that *T. bicolor* abundance decreases with elevation in Czech beech forests suggesting abiotic conditions restrict the range of the beetle. Abiotic conditions also restrict fungal distributions and prevalence suggesting there may be similar effects of elevation. For example, temperature can affect which fungi beetles develop with and which are acquired prior to dispersal [34, 38]. We found no such effect here. Similarly, because *T. bicolor* prefers sun-exposed wood and reaches highest population densities in extensive, mature forests while declining toward more open areas [7], we expected the treatments that altered canopy cover and deadwood exposure may influence its mycobiome. However, we detected no significant effect of treatment on mycobiome composition for either beetle species. Within BETA-FOR, canopy cover significantly affected deadwood fungal diversity in beech, though host tree species identity was overall the stronger driver of diversity across tree species [20]. Our results show no comparable influence of treatment on beech bark beetle mycobiomes, despite this structural effect on deadwood fungi. The coarser treatment grouping, combined with the broader unbalanced sampling across our design limits our ability to detect the effects of specific treatment types.

This hierarchical design, from forest patch (0.25 ha) to district (10–20 ha) to forest site to region (spanning climatic gradients), represents the first landscape-scale field study of bark beetle mycobiome diversity in temperate forests. The regional effect we detected did not hold up under sensitivity checks and largely reflects uneven sampling rather than a true regional driver. Forest site, however (selected to avoid the pseudo replication characteristic in treating single-site regions as independent replicates) remained a significant driver of composition, as did geographic distance between patches. We therefore conclude that mycobiome composition is spatially structured, but at a finer scale than region captures, rather than being uniformly weak across all spatial scales in our design.

After colonization on the patches, all logs were brought to the same facility and incubated in emergence traps for one year. Consequently, late-stage development occurred under partially standardized conditions that may have homogenized mycobiomes among regions, similar to how shared environments and sampling designs can generate what appear to be core microbiomes and lead to a reduction in spatial structure [26]. However, initial colonization and early larval development took place in the source forest patches, so the communities we sampled should still reflect realized within-log assemblages shaped by local field conditions rather than by the emergence trap facility alone.

We extracted DNA from crushed whole adults, including fungi from both inside and outside the beetles. Beetles were not surface sterilized and since this was a field-based study spanning six regions rather than a controlled laboratory setup, logs were exposed under natural conditions without controls to track log colonization, emergence traps were open to ambient air rather than sealed. This broad sampling most likely contributes to the dominance of ubiquitous saprotrophs and yeasts in our dataset while still capturing fungal associates that persisted through development inside the log. Since beetles were not surface-sterilized and emergence traps were open to ambient air, we cannot determine whether a given fungal taxon originated from the log, the trap environment, or the beetle itself, although many of the taxa recovered are known to be insect-vectored. This affects the biological interpretation of individual taxa rather than the compositional comparisons central to this study, since all samples were subject to the same field and trap conditions. Individual source trees were also not tracked, so logs within a forest site may have originated from the same or from few trees. On four patches, both beetle species emerged from the same log, meaning these paired samples share the same breeding conditions. Since host tree identity is a strong driver of deadwood fungal communities [20], this could contribute to the within-site similarity in mycobiome composition we observed. More generally, because our design used beech logs exclusively, we cannot rule out that host tree species identity, rather than the absence of true effects of region, elevation or beetle species, underlies the broad similarity we found across these predictors. Future work sampling additional host trees such as hornbeam and oak alongside beech may help disentangle this. We were also unable to include microclimate as a predictor because temperature and humidity were not recorded during log exposure, and within-log fungal communities have not yet been processed for this dataset. As poikilotherms, beetles and their associated fungi are sensitive to temperature, which affects fungal growth, sporulation and symbiont acquisition before dispersal [34, 38], highlighting the need to pair beetle mycobiomes with microclimate data in future work.

Several recurring fungal species have unknown ecological roles. Common deadwood saprotrophic macrofungi such as *Hypholoma fasciculare* and *Coprinellus micaceus* were frequent in both beetle species, as well as the yeast-like saprotrophic fungus *Aureobasidium pullulans.* Environmentally ubiquitous, wind-dispersed saprotrophs like *Cladosporium*, *Penicillium* and *Aspergillus* that can also be dispersed secondarily by insects, were also common with both beetle species. *Yamadazyma* spp., known gut symbionts of bark beetles (e.g., [29]), formed a main component of the mycobiomes of both beetle species, occurring in many samples and sometimes reaching high relative abundance. *Vishniacozyma victoriae*, a ubiquitous yeast that is found in the soils of temperate deciduous forests [21], was present in over a quarter of the samples. Occasionally, reads from macrofungi and a lichen (e.g., *Phallus impudicus*, *Physcia stellaris*) dominated individual samples, highlighting that these beetles can be vectors for diverse environmental fungi. ITS-sequencing can underrepresent *Ophiostoma* species known as beneficial symbionts of many bark and ambrosia beetle species, due to primer and amplification biases [12]. Further studies are needed to show if the almost complete absence of this genus from the samples (i.e., only one sample contained an *Ophiostoma* sp.) reflects natural absence or is a methodological artifact. *Beauveria*, a known bark beetle entomopathogen, was present only in two samples, consistent with a facultative or incidental association. While *Geosmithia* is one of the few fungal associations previously observed for both *E. fagi* [17] and *T. bicolor* [40], in our dataset the genus occurred at comparable levels in both beetle species, but the identified species *G. cnesini* was found almost exclusively in *E. fagi*. For *E. fagi*, the relative abundance of *G. cnesini* was highest in the three Saarland patches, though the species was also detected at lower relative abundances in two additional *E. fagi* samples from other regions. *Geosmithia* species are known to tolerate high temperatures and dryness compared to other fungi, because they typically associate with bark beetles colonizing thin and dying branches [16]. Our findings support the idea that many fungal associates of bark beetles are ecologically flexible, facultative symbionts, rather than obligate symbionts. More detailed investigations of individual galleries and transgenerational analyses of beetle mycobiomes will be needed to test if there are specific, vertically transmitted species hidden among the mostly flexibly associated fungi [33]. Our data did not reveal dominant species among those known as symbionts in other bark beetles, but our study spanned multiple regions and two beetle species. A future study focused on a single species at a single site may be better positioned to detect such symbiotic relationships.

## Conclusion

Our results showed that the beech bark beetles *E. fagi* and *T. bicolor* are associated with a diverse and nearly identical fungal community of saprotrophs and yeasts that are ubiquitously known from forests, with the exception of *Geosmithia cnesini*, which occurred almost exclusively in *E. fagi*. Broad landscape-scale gradients such as region, treatment, and elevation only weakly influenced these associations, while mycobiome composition was more consistently structured at the finer scale of individual forest sites and geographic distance, highlighting that regional-scale sampling designs risk missing real spatial structure in beetle mycobiomes. Future studies could expand on these findings by increasing sample size per forest site and including data on other potentially influential factors such as local microclimate data and individual host tree mycobiomes. More detailed studies at the level of individual beetle galleries are needed to reveal whether obligate, species-specific fungi are hidden within the broader diversity of fungal associates in these mycobiomes. This possibility is raised both by the host-specific pattern we found in *Geosmithia* and by the known limitations of ITS-sequencing for detecting *Ophiostoma*, a genus that includes obligate symbionts in other bark beetle systems. Far less is known about angiosperm-colonizing bark beetles than systems associated with conifers. Our findings suggest that flexible, saprotroph-dominated associations rather than obligate symbionts, may be typical of these beech bark beetle species.

## Supplementary Information

Below is the link to the electronic supplementary material.

Supplementary file1 (DOCX 5985 KB)

## Data Availability

Sequence data are available in the NCBI Sequence Read Archive under accession PRJNA1538716.
