# Supplementary material for: The Ecology of Beech Bark Beetle Mycobiomes: Similar Fungal Composition Despite Host, Elevation and Site Gradients
Source: Microb Ecol. 2026 Nov 4;89:202. doi: 10.1007/s00248-026-02910-3 (PMC13740426; doi:10.1007/s00248-026-02910-3)
Supplement: Supplementary file 1 [file 248_2026_2910_MOESM1_ESM.docx]

**Supplementary Material**

*Figure S1:* *iNEXT rarefaction and extrapolation curves for fungal ASV diversity in E. fagi and T. bicolor samples. The vertical dashed line marks the rarefaction threshold of 1000 reads per sample used in analyses, indicating that dominant components of the mycobiome (q = 1, q = 2) are well sampled, while total richness (q = 0) remains under sampled.*

*Table S 1: PERMANOVA comparison of rarefied vs. proportion-normalized data (n = 63). Bray-Curtis dissimilarity; 9999 permutations. R^2^ and p-values are nearly identical across both normalization approaches. This confirms results are not dependent on rarefaction.*

*Figure S2: Raw alpha diversity indices by beetle species (E. fagi and T. bicolor). Points show individual samples (*E. fagi*, n = 25;* T. bicolor*, n = 38). Boxplots show median and interquartile range. P-values from Kruskal-Wallis tests are shown in each panel. These indices are provided for comparison with studies not reporting Hill numbers.*

Figure S 3: Fungal genus composition per individual sample (n = 63). Bars show relative abundance per sample, faceted by beetle species. This shows the 18 most abundant genera by mean relative abundance across all samples. All remaining genera are pooled as "Other”.

Table S 2: Homogeneity of multivariate dispersion (BETADISP; 9999 permutations) for each grouping variable. All p-values are non-significant (p > 0.18), indicating comparable within-group variability across levels of management, region, beetle species, and forest site.

Table S 3: PERMANOVA results for beetle mycobiome composition by region for the full dataset and two sensitivity checks: excluding the two most thinly sampled regions (Hunsrück, Lübeck), and a direct pairwise comparison between the two best-sampled regions (University Forest, Bavarian Forest). The regional effect present in the full dataset (R² = 0.105, p = 0.026) is no longer significant once the two regions are excluded or when the two best-sampled regions are compared directly, showing this effect is not robust and does not represent a reliable region result.

Table S 4: Multiple regression on distance matrices (MRM) predicting mycobiome dissimilarity. The full model output with treatment dissimilarity, geographic distance, and elevation distance as predictors (9999 permutations). Geographic distance was the only significant predictor (coefficient = 0.00997, p = 0.011). Treatment and elevation distance were not significant. The overall model was significant (R^2^ = 0.022, p = 0.033), showing that geographic distance explains a small but significant amount of variance in mycobiome dissimilarity independent of the region or forest-site groupings.

Table S 5: PERMANOVA testing the effect of beetle species on mycobiome composition. Species tested alone and within the full model (species + treatment + elevation + region). Beetle species identity was not a significant predictor of mycobiome composition in either model (species-only: R^2^ = 0.025, p = 0.052; full model: R^2^ = 0.022, p = 0.119), rejecting H1.
